# Differences in perceived causes of childhood obesity between migrant and local communities in China: A qualitative study

**DOI:** 10.1371/journal.pone.0177505

**Published:** 2017-05-17

**Authors:** Bai Li, Rong Lin, Wei Liu, Jingyi Chen, Weijia Liu, KarKeung Cheng, Miranda Pallan, Peymane Adab, Laura Jones

**Affiliations:** 1 Institute of Applied Health Research, College of Medical and Dental Sciences, University of Birmingham, Birmingham, the United Kingdom; 2 Faculty of School Health, Guangzhou Centre for Disease Control and Prevention, Guangzhou, Guangdong Province, China; 3 Research office of health education, Guangzhou Health Care Promotion Centre for Primary and Middle Schools, Guangzhou, Guangdong Province, China; Universita Cattolica del Sacro Cuore Sede di Roma, ITALY

## Abstract

In developing countries, obesity traditionally affectsmore affluent children, butis spreading to a wider social group. Understanding the perceivedcontributors can provide valuable insights to plan preventive interventions. We exploreddifferences in the perceived causes of childhood obesity between local and migrant communities in a major Chinese city. We conducted 20 focus groups (137 parents, grandparents, school teachers) and 11semi-structured interviews with school Principals from migrant and local communities in Guangzhou. Data were transcribed and analysed using a thematic approach. We found that Lack of influence from grandparents, who were perceived to promote obesogenic behaviorin local children, fewer opportunities for unhealthy snacking and less pressure for academic attainment leading to moreactive play were interpreted as potential “protective” factors among migrant children. Nevertheless, two perceived causes of obesity were more pronounced in migrant than local children: lack of parental monitoring after-school andunsafe neighborhoods limiting physical-activity. Two barriers that restricted child physical activity were only found in the migrant community: limited home space, and cultural differences, inhabitinginteractive play with local children. Future interventions should consider uniquedeterminants of obesity in children from different social backgrounds, with tailored strategies to prevent further rise of the epidemic.

## Introduction

Following economic reform in the late 20^th^ Century, China has undergone rapid socio-economic transition[1]. This has resulted in accelerated urbanization as well as rural-to-urban and interprovincial migration, with large cities such as Beijing, Guangzhou and Shenzhen being the key migrant destinations[2–4]. Alongside this, the impacts of migration on health are becoming an important area for research and policy development. The health challenges faced in China are similar to those in other transitioning societies including an increasing burden of lifestyle related non-communicable diseases(NCDs)[5–6]. However, the majority of current migration health research has concentrated on infectious or transmitted diseases, mental illness, occupational health and healthcare system reform[7–8], with little focus on NCDs such as obesity.

Childhood obesity is a global public health crisis, linked to physical and psychological morbidity in children, and contributes to increasing NCDs and premature mortality in adulthood[9]. The most recent nationally representative study estimated that 15% of Chinese childrenand adolescents (aged 7 to 18 years) were overweight or obese, representing 30.43 million individuals. However, there is wide disparity in prevalence, with relatively low rates in rural areas (8.2% and 5.2% among boysand girlsrespectively), compared with prevalence rates that are similar to developed countries in large urban centres (32.6% and 19.1%)[10]. Of note, the rate of increase in the prevalence of childhood obesity in mainland China exceeds the trends observed in other countries, and over the last two decades, the rate of increase is most marked among boys in rural areas[11]. With increasing rural-urban migration, these migrant children are particularly susceptible to obesity. In a recent cross-sectional study undertaken in the city of Guangzhou (n = 9917, children aged between 5 and 12 years), we found that 14.3% of migrant children were either overweight or obese, although the prevalence was higher (20%) among local children[12]. Several studies have investigated the health impacts of migration on children whose parents have migrated. However, the focus has primarily been on the physical and psychological status of left-behind children[13–16]. The health of children who migrate with their parents has rarely been explored. Onecross-sectional study(n = 3368) comparing the dietary behaviorsof migrant and local adolescents (age 11–18 years) in the city of Shenzhen found that migrant adolescents were more likely to eat meals away from home, skip breakfast and to consume “street food” than local adolescents. Migrant adolescentsalso consumed significantly less fruit and vegetables[17].

Given the fast-moving socio-economic landscape and increasingly diverse urban population, effective control and prevention of childhood obesity in urban China requirescustomised intervention strategies. The guiding principles for the World Health Organization’s report of the commission on Ending Childhood Obesity advocates that governments should ensure equitable coverage of interventions particularly for vulnerable population groups who are at high risk of the double burdens of malnutrition and developing obesity[18]. In China, these groups include the migrant communities. Undoubtedly, an important step towards the prevention of the rise of the obesity epidemic among migrant children is exploring and understanding the perceptions of key stakeholders regardingthe causes of childhood obesity. To our knowledge, there are no qualitative studies which have explored this issue. Therefore, as part of a larger qualitative study that aimed to inform the development of culturally relevant childhood obesity prevention interventions in urban China, this sub-study focused on exploring the differences in perceived causes of childhood obesity between local and migrant communities living in a major city in China. Such insights can highlight shared, as well as unique perceived contributors to childhood obesity, and thus enable tailoring of preventive interventions to the needs of specific communities.

## Materials and methods

### Design and setting

This interpretivist qualitative study was conducted in a large southern city of China, Guangzhou, which is a major destination for migrant workers and their families[19]. There are approximately 10 million migrants living in Guangzhou and among the migrant parents, 70% have migrated with their children. Migrants accounts for 38% of the city’s total population and the figure is increasing annually[20–21].

### Participants and procedures

Participants were drawn from families and staff in primary schools in Guangzhou. The city has two main types of school. The majority(local schools)are state-funded and managed by local government, providing schooling only for local residents. The other (migrant schools) are privately run by students’ tuitions, where children of migrant families attend. We identified a range of relevant participants/stakeholderswithin the schools as having potentially important views and experiences related to childhood obesity, including families (parents and grandparents) and staff (principals, class-level teachers, physical education (PE) teachers). The Guangzhou Education Bureau provided consent to contact all (N = 318) primaryschools (both local and migrant)in five urban districts of Guangzhou. In each district schools were stratified as local or migrant. From these, one migrant and two local schools were randomly selected (using a random number generator) and invited to participate. Two of the migrant schools declined participation. A further three local schools were purposively sampled to provide a wider range of type of school in terms of location, size and social mix of the pupils. In each of the selected schools, agreement to approach potential participants was first obtained fromthe school principals, and thenthe school doctor or nurse helped to identify andinviteparticipants within their school community. The principals from all 16 schools sampled were invited via letter totake part in a one to one interview, and 11 of these accepted. Focus group participants were purposively sampledwith a 3:2 ratio for local to migrant schools, with separate meetings held for each identity group (i.e. parents, grandparents, class-level teachers& school nurses/doctors, and PE teachers), within local or migrant communities. Sampling of school staffaimed to achieve an equal gender balance with a representative range of ages. Parents and grandparents were invited by letter delivered through school by their child, and sampled to represent a balance in terms of the child’s school year (grade 1–5), sex and weight status.

This study did not aim to generate a theory and so data saturation is less relevant[22]. Our pragmatic sampling strategy sought to identify and recruit a maximum diversity sample of participants within each stakeholder group. This facilitated the exploration of diverse views within the available time and resources. Based on our previous qualitative data collection experience, we planned to conduct up to five focus groups with each of the four identity groups. In addition, we sought to interview up to 16 school principals (one from each of the participating schools).

Ethical approval was obtained from the Life and Health Sciences Ethical Review Committee at the University of Birmingham (reference ERN_13–1519). A local ethical approval was also granted by the Ethical Committee of Guangzhou Center for Disease Control and Prevention.

### Data collection

Data collection took place between October 2013 and January 2014. All participants had the study explained to them by the research team, and were given an opportunity to ask questions. Following this each participant then provided written informed consent prior to the commencement of data collection.

Semi-structured one to one interviews with the school principals took place in school meeting rooms using a discussion guide (Box 1) which was developed by the research team to explore their understanding of what contributes to childhood obesity in their community, and their views on how this could be prevented. The guide was used to ensure consistency between and within interviews/focus groups and explored perceptions of the causes of childhood obesity. Interviews lasted on average 55mins (range 45 to 70 minutes), were conducted in Chinese by one of three researchers (one Chinese speaking female, one Chinese speaking male, one mixed Chinese-Cantonese speaking female all of whom had received training from experienced qualitative researchers in qualitative data collection methods), and were audio recorded with consent.

Focus groups were chosen as the data collection method to help facilitate group interaction and promote discussion. Focus groups were held in a suitable room within the schools using the same discussion guide as that used in the interviews. The guide remained flexible and was reviewed after each interview/focus group to allow new ideas and interesting insights to be pursued throughout the data collection period. Groups had an average of 7 participants (range 4to 11) and lasted on average 65 minutes (range 40 to 80 minutes). Three moderators (and three assistant moderators divided into three groups, ran all focus groups). All groups were conducted in Chinese and audio-recorded with consent.

**Box 1. Topic guide for focus group and interview**

What do you believe are the most important factors contributing to increasing overweight among Chinese children nowadays?

Do you have any suggestions for how we might overcome the problem(any solutions)?

What could bepractically done to prevent Chinese children from becoming obese?

Do you have any suggestions for how we could get children to do more exercise / not spend too long doing sedentary activities?

How could we increase children’s activity levels–what type of activities, where and when could these take place, and how do you think this could be organized?

Do you have any suggestions for how we could improve children’s diet and encourage them to eat a more balanced and healthy diet?

What could be done by family members, teachers or others to support healthy eating in children?Where and when could such activities take place?How could they be organized?

### Data analysis

Data were analyzed using inductive thematic analysis based on the method proposed by Braun and Clarke[23]. Both the interviews and focus groups were guided by similar topic guides and so this allowed us to undertake an inductive thematic analysis across the whole data set (31 transcripts in total). The analytic process was iterative and took place concurrently with data collection. Audio recordings were transcribed clean verbatim in Chinese and then translated in to English with back translation of 2 randomly selected transcripts to check translation quality and validity. The data was primarily analysed in its original language (Chinese) by two native Chinese authors. Translation and back translation were conducted by two different professional translation companies. Discrepancies between the original Chinese transcripts and the back translated Chinese transcripts were checked by our Chinese authors. Minor discrepancies were found for 15 out of 676 original paragraphs of transcripts (2.22% error rate) but they did not alter the meanings of the original transcripts. The translated English transcripts allowed non-Chinese authors to participate in ongoing discussion and finalisation of the study results in the data analysis process described below.

Transcripts were read repeatedly to aid familiarization and allow the two analysts (BL and RL) to become immersed in the data. This facilitated the generation of preliminary codes and themes which was supported by NVivo10 software. Three of the most concept-rich transcripts were then independently coded by BL and RL and additional interpretations were incorporated into the code book. BL then applied the coding frame to the remaining transcripts and developed a working codebook in English which was reviewed by three members of the research team (MP, PA, LLJ). LLJ provided further ongoing support to BL to develop the thematic code book to specifically explore migrant and local views. Divergent cases were explored and reported in the results where appropriate. Our systematic and inductive analysis was significantly strengthened by multiple coders who themselves brought varied but valid perspectives to identify unique codes and themes within the data[24]. This provided an important opportunity to explore different explanations and interpretations of the data. Discussions continued within the team until agreement on the themes and interpretation of the data was reached, thus ensuring thoroughness, rigour and ultimately transparency in our analysis.

## Results

### Participant characteristics

A total of 148 participants were involved in the study (Tables 1 and 2): 137participants (44male) from 16 primary schools (3migrant schools) took part in 20focus groups (6in migrant communities); 11school principals (2 frommigrant schools)participated inone-to-one interviews. There wereno grandparent focus groups in the migrant communities, as grandparents generally had not migrated with the family. Although the focus groups were separated by identity groups, some school staffwere also parents and discussed their experiences and beliefs from a parent perspective, as well as within their capacity as teachers.

**Table 1:** Summary of the characteristics of study participants by type of residency status.

	Local communities (Total N = 104) N (%)					Migrant communities (Total N = 44) N (%)			
	Parents	Grand- parents	Class level head teachers	PE teachers	School principal	Parents	Class level head teachers	PE teachers	School principal
**Sex**									
Male	9(40.91)	15(45.45)	0	8(38.10)	2(22.22)	2(13.33)	3(23.08)	7(50.00)	2(100.00)
Female	13(59.09)	18(54.55)	19(100.00)	13(61.90)	7(77.78)	13(86.67)	10(76.92)	7(50.00)	0
**Age**									
60 or older	0	32(96.97)	0	0	0	0	0	0	0
50–59	2(9.09)	1(3.03)	0	7(33.33)	1(11.11)	1(6.67)	0	1(7.14)	1(50.00)
40–49	8(36.36)	0	8(42.11)	8(38.10)	8(88.89)	2(13.33)	1(7.69)	1(7.14)	0
30–39	12(54.55)	0	10(52.63)	6(28.57)	0	9(60.00)	2(15.38)	2(14.29)	1(50.00)
20–29	0	0	1(5.26)	0	0	3(20.00)	10(76.92)	10(71.43)	0
**Education level**									
No education or up to middle school	3(13.64)	20(60.61)	0	0	0	9(60.00)	0	0	0
High school	5(22.73)	9(27.27)	0	0	0	1(6.67)	0	0	0
University	14(63.64)	4(12.12)	19(100.00)	21(100.00)	9(100.00)	5(33.33)	13(100.00)	14(100.00)	2(100.00)
**Occupation**									
House work	6(27.27)	1(3.03)	0	0	0	5(33.33)	0	0	0
Unemployed	1(4.55)	0	0	0	0	3(20.00)	0	0	0
Retired	1(4.55)	25(75.76)	0	0	0	0	0	0	0
Manager/Director	2(9.09)	1(3.03)	0	0	9(100.00)	0	0	0	2(100.00)
Professionals (e.g. teacher and doctor)	3(13.64)	1(3.03)	19(100.00)	21(100.00)	0	3(20.00)	13(100.00)	14(100.00)	0
Service industry worker	7(31.82)	1(3.03)	0	0	0	3(20.00)	0	0	0
Agriculture, forest, livestock farming, and water conservancy industry worker	0	2(6.06)	0	0	0	0	0	0	0
Unknown/not reported	2(9.09)	2(6.06)	0	0	0	1(6.67)	0	0	0

Figures represent numbers and proportions of participants in each category of sex, age, education level and occupation, by types of residency. N = number; % = percentage

**Table 2:** Composition of local and migrant participants in each stakeholder identity group.

	Focus groups				Semi-structured interviews
	Parents	Grandparents	Class level head teachers	PE teachers	School principal
**Number of Focus groups Orinterviews (%)**					
Local	3(60.00)	5(100.00)	3(60.00)	3(60.00)	9(81.80)
Migrant	2(40.00)	0	2(40.00)	2(40.00)	2(18.20)
Total	5(100.00)	5(100.00)	5(100.00)	5(100.00)	11(100.00)
**Number of participants per group (total)**					
Local	8,7,7(22)	6,11,4,6,6(33)	6,5,8(19)	5,8,8(21)	
Migrant	6,9(15)	0	6,7(13)	7,7(14)	
Total	37	33	32	35	

Figures represent number and proportion of participants in each type of residency, by types of stakeholders. N = number; % = percentage.

### Core themes interpreted

**Fig 1:**
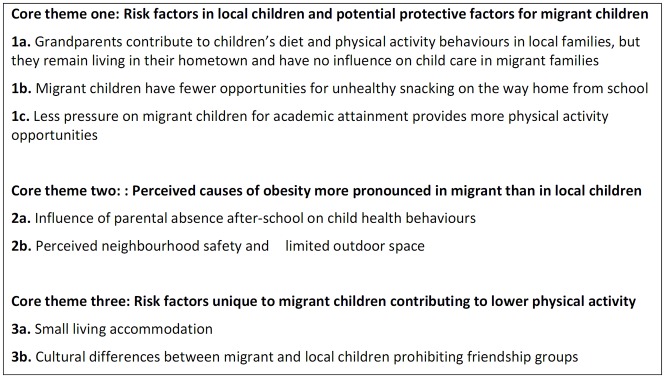
Summary of interpreted core and sub themes.

Three core themes with seven sub themes were interpreted within the data (Fig 1) which highlight key differences in the perceived causes of obesity between local and migrant children. In particular, three potential protective factors for obesity among migrant children were interpreted (core theme 1). Two shared perceived causes of childhood obesity were more pronounced in migrant communities compared with local communities (core theme 2). We also interpretedtwo perceived causes of childhood obesity that were only highlighted by migrant communities (core theme 3).

#### Core theme one: Risk factors in local children and potential protective factors for migrant children

**1a**. **Grandparents contribute to children’s diet and physical activity behaviors in local families, but they remain living in their hometown and have no influence on child care in migrant families**

All participating school principals and teachers from migrant communities noted that grandparents of the children living in migrant families usually remained in their hometown. Thus, unlike the local community, issues related to cross-generation child care were not discussed by migrant families.

‘I really agree thatgrandparents should be an important target for child obesity intervention among local communities but this is not applicable for our children (i.e. children attending migrant schools) due to the unique family characteristic…grandparents usually remain in their hometown so they do not live with the children’

(Migrant school principal, principal Int 13).

In contrast, a recurrent theme in all groups from local communities was around the child care provided by live-in grandparents. Having one or more grandparents living in the household seemed to be very common in local families.

‘Now, both fathers and mothers work…so cross-generation family structure and grandparent-led child care are very common’

(Local school class teacher1, class teacher FG 8).

‘My husband and I go to work in the day so child care is the responsibility of the elderly’

(Local school principal, principal Int 1).

Although live-in grandparents often took considerable responsibilities in caring for theirgrandchild, they were widely blamed fortheir contribution to childhood obesity. Such discussions were concentrated ongrandparents’ preference for a fat child, and how they overfed and promoted excess weight gain among the young.

‘They (grandparents) have out of date concepts, they believe that being fat is a symbol of health and being slim is a symbol of under nutrition or bad health… you can see the majority of overweight children are looked after by grandparents…busy parents do need help from their parents so they often struggle to express their worries let alone challenging their parents…many grandparents are proud of their ‘big’ grandchildren and often say that the ‘big’ child is the outcome of their successful hard work’

(Local school mother 2, parent FG 11).

‘To them (grandparents), being fat is a very lucky thing; it represents a wealthy and contented life’

(Local school mother 2, parent FG 11).

‘It’s very obvious, our child always puts on weight during school holidays as he stays at his grandparents’ place and will return to his normal weight when he returns home during term time’

(Local school father3, parent FG 16).

The above perceptions werereinforced by grandparents, although participants in grandparent focus groups generally did not talk as much as other stakeholders about their child caring practice.

‘We (grandparents) have a common characteristic, we like to pamper our grandchildren…sometimes I know he is eating too much, but I am so happy to see him keep eating and getting bigger…we can’t ask the little one to stop eating if that’s what he likes to do…so we are a problem. I think the child’s obesity is directly related to us’

(Local school grandmother5, grandmother FG 7).

**1b**. **Migrant children have feweropportunities for unhealthy snacking on the way home from school**

Another perceived cause of childhood obesity as discussed by school principals and teachers from local communities was the common presence of unlicensed food traders close to school gates. This offered opportunities for unhealthy snacking among local school children.

‘Our students run fast to the unlicensed food traders and buy unhealthy snacks everyday as soon as they are allowed to leave the school. They are so popular…I have noticed some traders even allow children to buy on credit (e.g.to pay the next day) and accept advance payments (e.g. with a single 100 RMB pre payment, the paying kid and his/her friends can use the credits to purchase snacks until the 100RMB is run out)’

(Local school class teacher 6, class teacherFG 2).

In contrast the sale or consumptionof unhealthy food after school was not discussedas a problem for migrant communities. This was mainly because of the distance of school from the children’s homes and travel arrangements after school.

‘We don’t have the problem of food traders selling unhealthy food around the school gate because over 95% of our students live very far from the school so we use school buses to transport them between where they live and the school…they always come into and leave the school by bus so there are no chances for them to stop at the school gate to buy or eat snacks’

(Migrant school principal, principal Int 13).

Moreover, while children from local families are often picked up by parents or grandparents, children from migrant families were rarely accompanied by any family members on their way home. As illustrated below, having a family member at school pick-up presentsfurtheropportunities for unhealthy snacking after school.

‘We see grandmothers and grandfathers arriving at the school with cookies, eggs, milk etc…they don’t come with empty hands…so much food is given to the kids after school, so we expect that the amount of food our children are given is equivalent to 4 or more meals a day’

(Local school class teacher3, class teacher FG 2).

‘My child does not like to be picked up by me; he likes to have his grand-mum instead because my mum will come with his favourite snacks’

(Local school mother1, parent FG 16).

**1c**. **Less pressure on migrant children for academic attainment provides more physical activity opportunities**

All focus group and interview participants from local communities shared the belief that families and schools prioritized academic attainment over any other activities in the school, which was driven by the exam-focused social environment. They considered thisfocus on sedentary based homework and learning activities (on and off campus) limited the opportunity for children to engage in physical activity or free play.

‘The child doesn’t get enough exercise. When she returns home, it’s dinner time, after dinner, she starts doing homework, when she completes her homework, it would be very late so she goes to sleep immediately’

(Local school grandfather8, grandparent FG 7).

‘Children don’t do enough physical activities, in the weekends many of them go to math and English lessons’

(Localschool PE teacher7, PE teacher FG 14).

‘If we were not living in an education-oriented environment, if there was no study pressure and not so much homework, my child would have been able to enjoy different activities outdoors’

(Migrant school PE teacher 3, PE teacher FG 13, also a mother of a child in a local primary school).

In contrast, no participant from the migrant communities specifically talked about excessive homework or learning activities among their children. Therefore, the exam focused atmosphere that was believed to be a barrier to physical activity and promote obesity by stakeholders in the local communities did not appear to beperceived by migrant stakeholders. This observation was further confirmed by the recollections of a mother describing the very different experiences of her daughter who had recently moved from a migrant to a state-funded (local) school.

‘My daughter used to learn dance and she danced very well… she enjoyed various interests but now she does nothing other than studying…she is under great pressure…she does homework all the time and has no time for exercise…we are parents, we also feel lots of pressure… when we saw other parents sending their children to off-campus tutoring classes and saw their children performing better and better in exams, we followed…if other parents assigned additional homework to their children, we also followed…our daughter had never returned to her previous slim shape since she joined the new school’

(Migrant school PE teacher 3, PE teacher FG13, also a mother of a child in a local primary school)..

This story illustrated that local children often bear heavier study pressure and higher expectation to excel academically than their migrant counterparts. This in return can lead to less physical activity opportunities and consequent unhealthy weight gain.

#### Core theme two: Perceived causes of obesity more pronounced in migrant than in local children

**2a**. **Influence of parental absence after-schoolon child health behaviors**

A major and recurrent issue discussed by all participating parents and school staff from migrant communities was that migrant parents often worked long hours and remained at their work places in the evenings and at weekends.

‘Over 90% of migrant parents take night shifts and since there are no grandparents living together, the kids have to stay at home on their own in the evening’

(Migrant school principal, principalInt 15).

This meant they were unable to look after, monitor the children’s eating andother activities after school. Theseirregular working patterns were believed to impact on the children’s dietary and physical activity behavior and habits.

‘Because the parents have no time to do(physical activity) with the child, and the kid just sitting there, watching TV or playing games in the evening until washing and going to bed.’

(Migrant school mother 5, parent FG 10)

‘Nowadays the people, the adults are under pretty big work pressure, ah…Sometimes the adults come home from work and they themselves are too tired…Need some rest, have no time and energy to go running, skipping with the children.’

(Migrant school mother 2, parent FG 15)

‘Many migrant parents give money to their children to buy fast food for dinner…in the long term, an unhealthy diet can lead to obesity’

(Migrant school class teacher5, class teacher FG 10).

Lack of parental attention to healthy eating or failure to encourage and accompany children’s participation in physical activity, were not unique to migrant families. However, unlike migrant parents who were unable to influence children’s behavior due to irregular working pattern and workpressures, among the local community this was mainly attributed to low parental knowledge, skills, motivation or interest. Furthermore, there were participants from the local community who provided positive health-promotion parenting examples–something that was not mentioned in any focus groups or interviews undertaken in the migrant community.

‘It depends on the interest of a child’s parents, if the parents enjoy exercise, they will take their child to engage in sports; if the parents are not interested in exercise, even if they know how important exercise is, they wouldn’t necessarily do any sports with their child’

(Local school class teacher 5, class teacher FG 2).

‘Most parents are able to spend some time with their children at the weekend for exercise…for example many parents of my child’s classmates arrange group outdoor activities every Sunday because we don’t work on Sundays…a big group of parents and children going to a park, playing football or badminton…a variety of activities’

(Local school father1, parent FG 16).

‘Even for very busy parents, they should be able to do some exercise with their children in the weekends’

(Local school father 5, parent FG 16).

‘As long as adults (parents/grandparents) have the rightknowledge and skillsabout child-feeding, the healthy eating behaviors of their child will be well managed and controlled at home’

(Local school class teacher 1, class teacher FG 2).

**2b**. **Perceived neighbourhoodsafety and limited outdoorspace**

Concerns about neighbourhood safety was a theme across both communities, but was more prominent in migrant groups. Many local families lived in purpose built community housing, which are flats or houses developed and built by property firms or large employers with green areas and outdoor gyms. Migrant families on the other hand, typically rented small living spaces in resource-deprived locations. The problems of neighbourhoodsafety (busy traffic)and limited leisure space were recurrent topics discussed by parents and school staff from the migrant community.

*‘For families who don’t live in purpose built communities, children have very little space to play*’

(Migrant school mother2, parent FG 15).

*‘They (migrant children) will see busy streets as soon as they walk out from their residential building, so where can they play or run?*’

(Migrant school principal, principal Int 15).

*‘We (parents) are at work so can’t accompany our child to play outside our flat…with many fast cars moving around, we don’t feel it’s safe for a child to go outside on his own*’

(Migrant school father2, parent FG 15).

*‘For migrant families, lack of time is not the only problem. Migrant families live in rented flats close to busy roads, so migrant childrenhave little space to play or do physical activity around where they live*’

(Migrant school class teacher6, class teacher FG 10).

In contrast, only a few participating parents from the local community expressed concerns over neighbourhood safety (which focused on child trafficking rather than car traffic), or mentioned insufficient space for children to play near home. In fact, some local parents commented on the opportunities for physical activity and facilities available in their residential areas.

‘Why do I think my son does enough physical activity? In our community (purpose built employee residential community with leisure facilities and green areas), children go out to play in groups, Monday to weekend…everyday they engage in physical activity, there is no need for adults to motivate or supervise them…every time he comes home sweating so we have to change his clothes’

(Local school father1, parent FG 16).

#### Core theme three: Risk factors unique to migrant children contributing to lower physical activity

There were two issues that were only highlightedby stakeholders from the migrant community, both of which were believed to restrict physical activity opportunities in children. One was related tothesmall size of homes where migrant children typically reside and the other was about friendship building between migrant and local children.

**3a**. **Small living accommodation**

Small crowded living environments further exacerbated the perceived lack of neighbourhood safety in restricting physical activity opportunities for migrant children. In contrast, no participant from the local community mentioned the issue of insufficient space within the home.

‘The majority of migrant parents are unable to provide a spacious flat for their children…live in a very small rented flat…there may be one dining table, two chairs, one desk and nothing else…only local families who work for large organizations would have a large home’

(Migrant school class teacher 6, class teacher FG 10).

**3b**. **Cultural differences between migrant and local children prohibiting friendship groups**

Where migrant and local families lived in the same area, language and cultural barriers wereperceived deterrents to migrant children participating in outdoor sports and activities. In one focus group with migrant parents, all participants strongly agreed that their children were excluded from such activities. This theme was not discussed in other groups, and no participants from the local community mentioned any problems of free play or friendship building among local children.

*‘Local children don’t play with our children; we are migrant families*’

(Migrant school mother2, parent FG 15).

*‘I encouraged my child to play with local children but it didn’t change anything, local children wouldn’t play with migrant children*’

(Migrant school mother 1, parent FG 15).

*‘My child never wants to play with the son of the landlord who is from Guangdong province…equally, the boy never wants to play with my son*’

(Migrant school father4, parent FG 15).

When the focus group facilitator explored perceived reasons, participants attributed this to different dialects and game rules that made migrant children’s active play with local children difficult.

‘We know and play different games…with the similargame, local people have their rules while we (migrant people) and our children have our own rules…different rules’

(Migrant school mother 1, parent FG 15).

‘…local children and our children speak different dialects…so basically can’t communicate properly’

(Migrant school mother3, parent FG 15).

## Discussion

### Summary of principal findings

We explored the differentperceived influenceson diet and physical activity behaviors which affect levels ofobesity in children amonglocal and migrant communities living in a major city in southern China. Whilst unhealthy eating, lack of physical activity and high levels of sedentary activities were implicated in all groups, factors that led to those behaviors differed betweencommunities. Childcare by overindulgent grandparents (offering food rewards and restricting physical chores in children), sale of unhealthy snacks byunlicensed food traders outside school and low priority for physical activity within an environment that emphasizes academic achievement were prominent influences in local families, but were not concerns among migrants. Other situations were more common among migrants. This includedrisk of unhealthy diet and long periods of sedentary time due to lack of parental supervision resulting from long working hours, and limited outdoor physical activity due to perceived lack of neighborhood safety. Yet other barriers to physical activity, including lack of indoor space, andcultural differences leading to exclusion from play opportunities with local children, were unique to migrant families. Our findings suggest thatthe increasing migrant child population is at significantrisk of rising levels of childhood obesity. The identified differences in contributors to diet and physical activity behaviors between migrant and local communities meanthat customized prevention programsshould be developed for the migrant population.

### Findings in relation to previous literature

The influence of grandparents and other informal carers on child eating behavior and weight status have been reported previously in both urban[25]and rural China[13]as well as in other parts of the world[26–28]. Grandparents are commonly involved in childcare in urban China[29], making them important targets for childhood obesity prevention interventions. However, their absence in migrant households provides an opportunity for development of more focused interventions with parents in these groups.

The belief among local families that the presence ofunlicensed food traders outside schools contributes tounhealthy eating and childhood obesity, echoes findings from a previous study[30]. Internationally, a review of the distribution of fast food outlets in relation to health outcomes concluded that there is an association between higher density of such retailers and lower levels of healthy eating and that there is a preponderance of such outlets in the vicinity of schools[31]. However, whilst some studies do show a relationship between higher concentration of fast-food outlets and obesity[32–33], methodological constraints limit drawing of firm conclusions[31]. Nevertheless, policies to limit fast food sale in proximity of schools and in residential neighbourhoods are being implemented in some countries[34] and could be considered in China.

The perceived influence of the wider neighbourhood environment (including lack of green space and traffic concerns) on obesity, which was prevalent in migrant communities, has been the focus of much observational research. A systematic review of such studies found that overall, greater distance to playgrounds, and perceived poor access to green space and leisure facilities were associated with higher obesity levels in children[35]. Studies examining the relationship between perceived neighbourhood safety and physical activity levels or obesity were mixed. In some migrant communities, the neighbourhood barriers to physical activity were further exacerbated by cultural differences that inhibited friendship formation with local children. An overview of studies confirmed the positive influence of friendship and peers on children’s physical activity levels[36]. A more recent US qualitative study discussed the importance of neighbourhood playmates infacilitating physical activity among children[37], and a UK observational study found that time spent outdoors with other children was an important source of after schoolphysical activity[38]. Thus overall, interventions to improve access to safe play areas outside of school may be useful for migrant communities.

The perceived influence of parental motivation, attitudes and behaviour on children’s diet and physical activity behaviours and risk of obesity is in keeping with evidence from observational and experimental studies. Narrativereviews have shown that the physical and social home environment contributes to children’s physical and sedentary activity levels[39–40]. Parental practices and role modelling are important in shaping children’s physical activity levels, particularly in their early years[40], whilst parental control of the home physical activity environment is important in reducing sedentary time as children get older[39]. Similarly, parental role modelling, behaviour and control have been shown to influence children and adolescents’ dietary intakes[41]. Whilst interventions to modify parental motivation to encourage health behaviours in children may be helpful among local families, this is less likely to be effective for migrant communities where parents are absent for longer periods. Instead, intervention with migrant parents needs to acknowledge and accommodate their long working hours.

### Strengths and limitations

To our knowledge, this is the first qualitative study to explore and identify differences in perceived causes of childhood obesity between local and migrant communities in urban China. This is a relatively large qualitative study involving 20 focus groups and 11 semi-structured interviews with a total of 148 participants. We have therefore explored the views of a range of key stakeholders who came from a variety of socio-economic backgrounds. This allowed perceptions from a broad perspective to be explored. Our findings offer new insights into possible explanations of socio-economic disparities in childhood obesity epidemic documented in previous literature. Our findings relating to the perceived causes of childhood obesity were aligned with documented risk factors. Each of the transcripts were initially read by two members of the research team and a proportion of the most concept rich transcripts were independently double coded to help ensure coding consistency and increase coding integrity. Coding and interpretation were discussed at regular intervals between the analysts, with LLJ and the wider research team. This helped to ensure that the interpretations were grounded within the data and facilitated clarification of core concepts from multiple perspectives and the development of shared understanding. There are however some limitations to this study. This is an international collaboration with only a minority of the team fluent in both English and Chinese. Data were collected and predominantly analyzed in Chinese with subsequent translation of codebooks in to English to allow the English researchers to support analysis and interpretation. Although robust methods were used for the translation there is potential for interpretive errors. We used thematic analysis to interpret our findings. Whilst this had the advantage of allowing us to summarizekey themes and highlight similarities and differences between identity groups and local versus migrant community participants, our findings are predominantly descriptive and limited by the absence of a theoretical framework for interpretation.

### Implications for future research and policy

Although childhood obesity is currently more prevalent among the local children compared to migrant children living in urban China, recent trends suggest a steeper increase in the latter group. Our study has identified several perceived causes of obesity that were either more pronounced in, or unique to migrant children living in a major Chinese city. The results highlight the need for the development of tailored interventions to prevent a significant rise in the prevalence of migrant children who are overweight/obese and the associated short and longer term health consequences. Thus future interventions for local communities should consider education for grandparents, enforcement of regulations limiting illegal food traders outside schools and school policies that re-balance a focus on academic outcomes with increased physical activity opportunities. Within migrant communities on the other hand, interventions should focus on supporting parents and provision of more physical activity opportunities outside of school.

## Conclusions

Many of the influences promoting obesogenicbehavior among local children require policy level interventions, such aslimiting the sale of unhealthy snacks at school gates or increasing the priority of physical education relative to academic education in schools. In contrast, for migrant children policy level initiatives should focus on improving access to safe play areas and green space. In both groups, preventive interventions that focus on education and skill building are likely to be important. However, among local families, targets for such intervention should include parents and grandparents, whereas parents and schools should be targeted for migrant children. Our findings highlight the importance of qualitative research in helping our understanding of the variety of influences on childhood obesity, and in helping to tailor interventions. Many of the perceived causes of obesity reported here are also described in studies internationally. However, without such detailed exploration, policy makers could not interpret the relative importance of different influences or how to customizeinterventions for specific populations.

Future research should focus on the development and evaluation of appropriate intervention strategies to prevent further rise of the obesity epidemicin China.
